# *Bordetella pertussis*-infected innate immune cells drive the anti-pertussis response of human airway epithelium

**DOI:** 10.1038/s41598-022-07603-8

**Published:** 2022-03-07

**Authors:** M. M. Kroes, A. Miranda-Bedate, R. H. J. Jacobi, E. van Woudenbergh, G. den Hartog, J. P. M. van Putten, J. de Wit, E. Pinelli

**Affiliations:** 1 Center for Infectious Disease Control, National Institute for Public Health and the Environment, Bilthoven, The Netherlands; 2 Department of Infectious Diseases and Immunology, Faculty of Veterinary Medicine, Utrecht University, Utrecht, The Netherlands; 3 Section Paediatric Infectious Diseases, Laboratory of Medical Immunology, Radboud Institute for Molecular Life Sciences, Nijmegen, The Netherlands

**Keywords:** Cell biology, Immunology, Microbiology, Pathogenesis

## Abstract

Pertussis is a severe respiratory tract infection caused by *Bordetella pertussis*. This bacterium infects the ciliated epithelium of the human airways. We investigated the epithelial cell response to *B. pertussis* infection in primary human airway epithelium (HAE) differentiated at air–liquid interface. Infection of the HAE cells mimicked several hallmarks of *B. pertussis* infection such as reduced epithelial barrier integrity and abrogation of mucociliary transport. Our data suggests mild immunological activation of HAE by *B. pertussis* indicated by secretion of IL-6 and CXCL8 and the enrichment of genes involved in bacterial recognition and innate immune processes. We identified IL-1β and IFNγ, present in conditioned media derived from *B. pertussis*-infected macrophage and NK cells, as essential immunological factors for inducing robust chemokine secretion by HAE in response to *B. pertussis*. In transwell migration assays, the chemokine-containing supernatants derived from this HAE induced monocyte migration. Our data suggests that the airway epithelium on its own has a limited immunological response to *B. pertussis* and that for a broad immune response communication with local innate immune cells is necessary. This highlights the importance of intercellular communication in the defense against *B. pertussis* infection and may assist in the rational design of improved pertussis vaccines.

## Introduction

The highly contagious respiratory disease pertussis, also known as whooping cough, is caused by the Gram-negative bacterium *Bordetella pertussis*. This disease only affects humans and is characterized by a severe persistent cough and leukocytosis^1^. Pertussis cases have been increasing in the last few decades despite high vaccination coverage, infecting an estimated 24.1 million individuals and causing 160,700 deaths in children younger than 5 years in 2014^2^. Several reasons for this reemergence have been suggested including pathogen adaptation, waning of vaccine-induced immunity and suboptimal induction of a protective type of immune response by the acellular pertussis vaccine (aP)^3–5^.

*Bordetella pertussis* is transmitted via aerosol droplets and upon infection attaches to the ciliated epithelium of the trachea and bronchia^1^. The airway epithelium forms a physical barrier and contains cilia that continuously beat in a coordinated manner to expulse mucus and trapped particles from the airways, a process termed mucociliary clearance. *Bordetella pertussis* damages the ciliated epithelium and disrupts mucociliary clearance by attaching to the ciliated cells and secreting virulence factors such as tracheal cytotoxin (TCT)^6,7^. This leads to pulmonary obstruction due to accumulation of mucus in the respiratory tract^1^. The airway epithelium is not only a physical barrier but it is a complex network of epithelial and resident immune cells that closely interact^8^. Sensing of the pathogen by the airway epithelium will initiate communication between this complex network of cells resulting in a host cell response to limit infection. The airway epithelial cells produce host defense peptides as well as chemokines and cytokines^9^. The chemokines will attract other immune cells to the site of infection and cytokines are involved in the activation of immune cells leading to an inflammatory response in the respiratory tract. Infection of the respiratory tract by *B. pertussis* results in the initial recruitment of macrophages and dendritic cells (DCs), shortly followed by an influx of neutrophils and natural killer (NK) cells^10^. *Bordetella pertussis* infection-studies in mice and baboons have shown the production of various cytokines and chemokines such as TNF, IL-1β, IFNγ, CXCL8, CCL2, CXCL9, CXCL10 and CXCL11 in the lung^11–13^, however, the contribution of the epithelial cells to these immunological responses remain unknown.

The currently used aP vaccine protects well against pertussis disease, however, it does not prevent colonization by, or transmission of *B. pertussis*^14^. This allows circulation of the bacterium in the vaccinated population. For the rational design of next generation vaccines that prevent colonization by the bacterium, better knowledge on the local immune response against *B. pertussis* in the human respiratory tract is essential. Despite extensive research on immune responses to *B. pertussis*^10^*,* little is known about how the initial immune response at the human airway epithelium is orchestrated. In the present study, we investigate the immune response to *B. pertussis* at the human airway epithelium using primary human airway epithelial cells (HAE) differentiated at air–liquid interface. The HAE response to this pathogen was characterized by assessing mucociliary transport, barrier integrity, transcriptomics as well as cytokine and chemokine secretion. As during natural infection the airway epithelium closely interacts with immune cells, we also investigated the possible regulatory role of other innate immune cells on the HAE response to *B. pertussis*. Our results indicate that HAE requires communication with other *B. pertussis-*exposed innate immune cells. We identified IFNγ and IL-1β as critical immunological factors for the initiation of a robust immunological response to *B. pertussis* by HAE cells. Additionally, we show that the chemokines produced by these HAE effectively recruit monocytes.

## Results

### Characteristics of the HAE infection model

**Figure 1:**
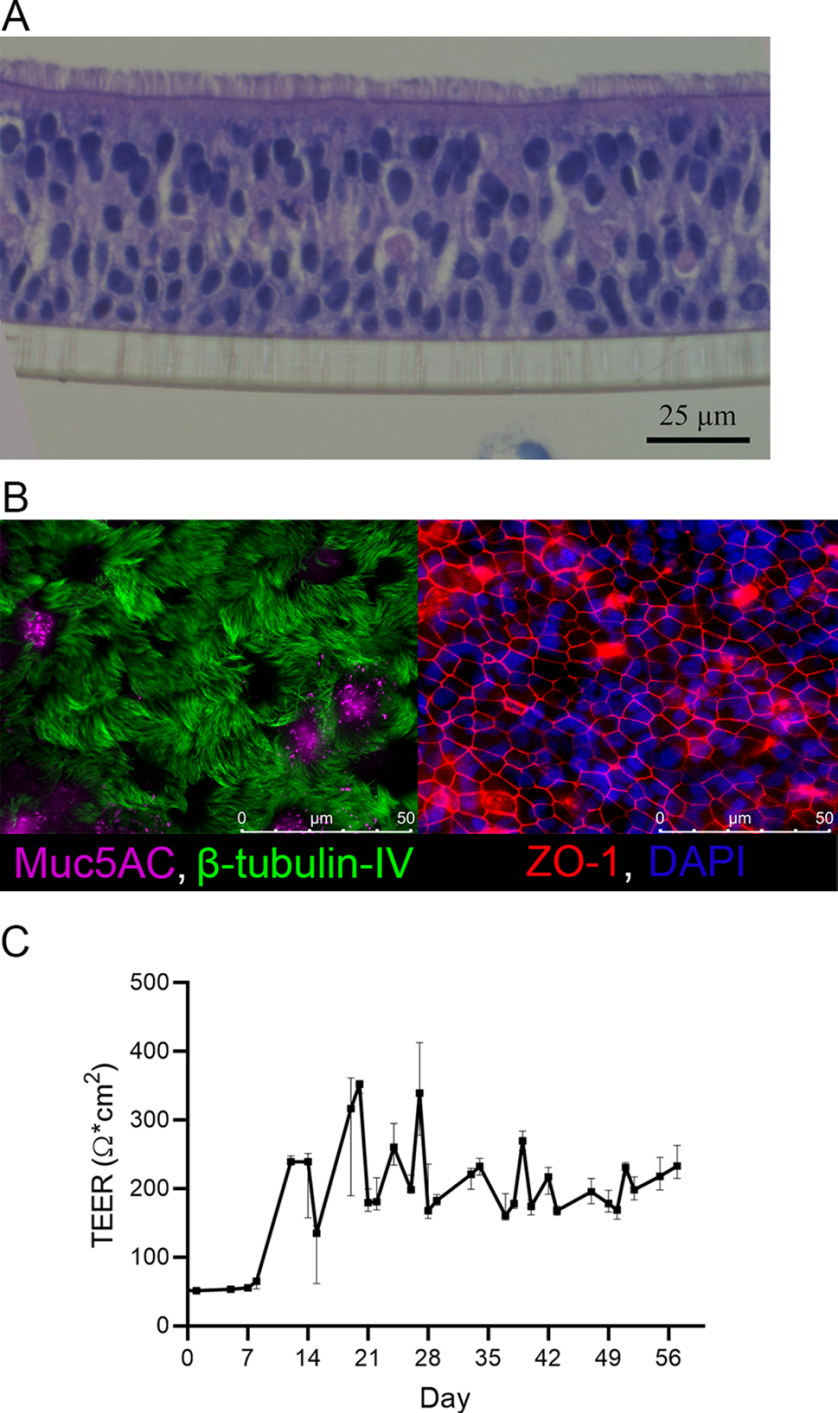
HAE cultures. (**A**) HE staining of HAE cultures differentiated for 7 weeks. (**B**) Fluorescence microscopy images of HAE cultures differentiated for 7 weeks and stained with (left panel) β-tubulin IV (Cilia, green) and Muc5AC (Mucus, pink) or (right panel) ZO-1 (Tight junction, red) and DAPI (Nuclei, blue). (**C**) TEER development during HAE culturing in time (days). Data are represented as median and 95% confidence interval of 4 different donors from 4 independent experiments.

*Bordetella pertussis* infects the human respiratory tract and in this study we set out to investigate early immune responses of the human airway epithelium to this bacterium. To this end we set up an in vitro model that closely represents the natural infection niche of *B. pertussis* by using primary human bronchial/tracheal airway epithelium cells. Culture of these cells on transwells and exposing them to air on the apical side for 6–8 weeks yielded well differentiated human airway epithelium (HAE). HE staining of cross-sectional slices of the cells after 7 weeks of differentiation revealed several cell layers of epithelial cells with protruding cilia on the apical side (Fig. 1A). The HAE cultures also produced mucus, which was visible as a viscous layer on top of the cells. Fluorescent microscopy on the HAE cultures revealed the presence of several mucus producing (Muc5AC^+^, Pink) and a large amount of ciliated (β-tubulin IV^+^, Green) cells which were connected by tight junctions (ZO-1^+^, Red) (Fig. 1B). To assess the barrier function of the HAE cultures we measured the transepithelial electronical resistance (TEER). Cultures from all donors used in this study showed significant increases in barrier function one week after air–liquid culture. Maximum TEER values were reached after three to four weeks and remained stable at ~ 200 Ω*m^2^ for the rest of the culture period (Fig. 1C).

### Disruption of barrier integrity and mucociliary clearance in *B. pertussis*-infected HAE cultures

**Figure 2:**
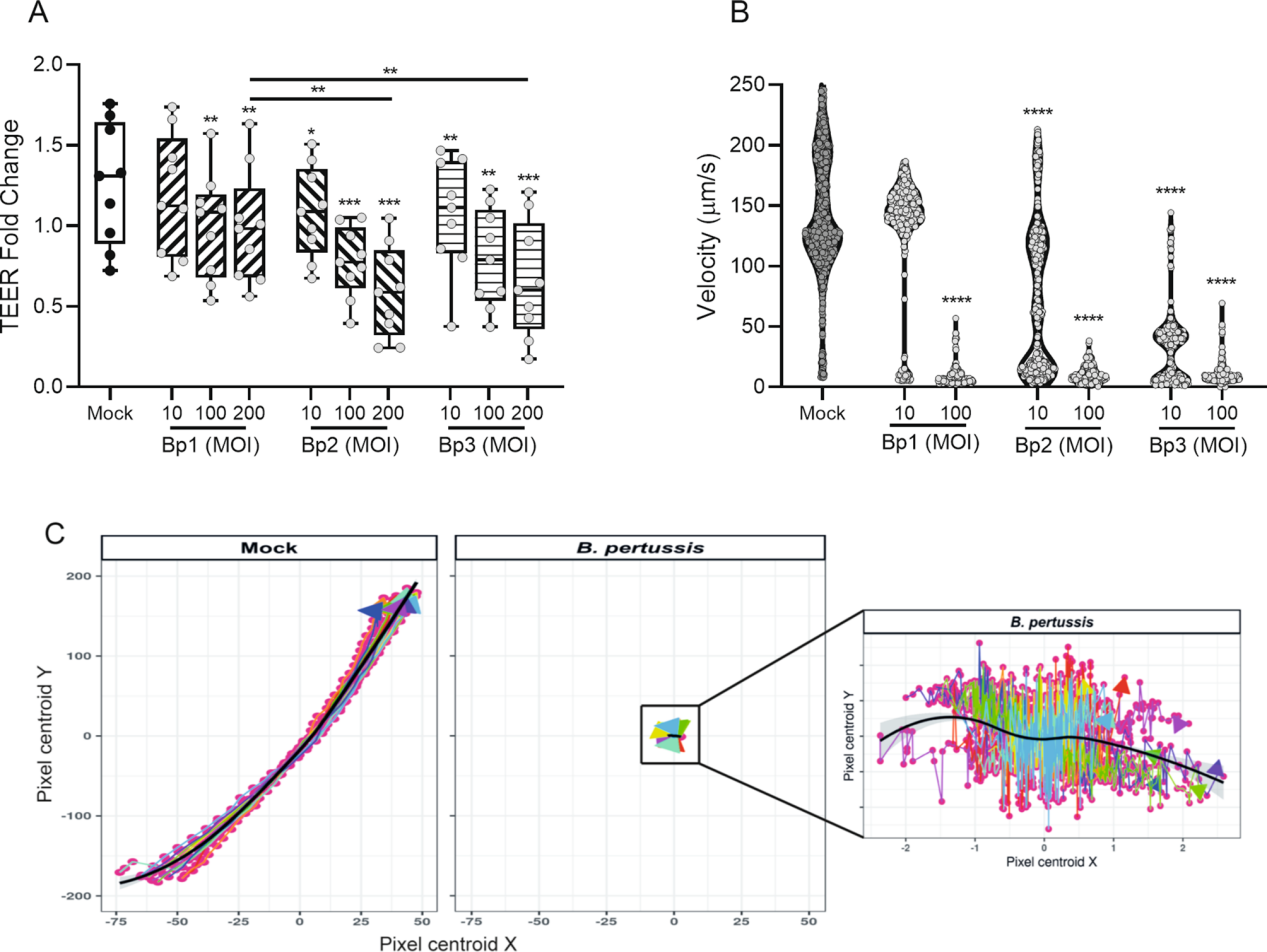
Barrier function and mucociliary clearance after stimulation with three different *B. pertussis* strains. (**A**) TEER of HAE cultures after 22 h in medium only (Mock, black dots) or with *B. pertussis* stimulation (grey dots) relative to the TEER prior stimulation. Data are represented as boxplots and the filling represents different *B. pertussis* strains used and individual dots are averages of triplicate measurements. The data shown are from 4 donors measured twice across 3 independent experiments. (**B**) Velocity of beads moving over the apical surface of HAE cells after a 22 h Mock (black) or *B. pertussis* (grey) stimulation. Data are represented as violin plots, dots represent individual bead trajectories. The data shown are from 2 donors from 3 independent experiments. Significance represented relative to Mock-treated HAE cells. *p < 0.05, **p < 0.01, ***p < 0.001, ****p < 0.0001. (**C**) Normalized directional bead displacement of Mock- (left panel) or *B. pertussis*- (middle and right panel) stimulated HAE cells. Individual dots represent individually detected beads and arrows indicate trajectory of beads, black line indicate average bead locations. Data shown are of one representative location in one Mock- or *B. pertussis*-stimulated HAE cells.

Two hallmarks of pertussis infection are cellular damage of the airway epithelial lining and disruption of the mucociliary clearance^1^. To assess whether these hallmarks can be mimicked in our HAE cultures, we inoculated the cells with three different live naturally circulating *B. pertussis* strains at various MOI. After 22 h of incubation we observed that all *B. pertussis* strains reduced the TEER in a dose dependent manner as compared to the Mock HAE cultures (Fig. 2A), indicating disruption of the epithelial barrier function by the pathogen. A significant difference in TEER disruption is observed between Bp1 and the other two strains at MOI 200. However, the basis for this variation remains unknown. Furthermore, we analyzed the ability of the HAE to transport beads over the apical surface of the cells by using fluorescent beads in combination with live imaging. Due to the damaging effect of a *B. pertussis* MOI 200 inoculation on the HAE cultures, only the lower MOIs were included in this analysis. Mock HAE cultures transported the beads with an average speed of 145 µm/s in a single direction (Fig. 2B,C, Movie S1, S2). In stark contrast, incubation with MOI 100 of any of the three *B. pertussis* strains completely disrupted the ability of the HAE cells to transport the beads over their apical surface (Fig. 2B, Movie S3, S4). Incubation of HAE with *B. pertussis* at this MOI resulted mostly in immobile beads with some limited randomly-oriented movements compared to the longer trajectory and one directional movement of the beads in the Mock HAE cultures (Fig. 2C). At a lower MOI of 10, strains Bp2 and Bp3 significantly reduced the speed of bead movement whereas strain Bp1 did not affect the HAE ability to transport beads (Fig. 2B). These data show that *B. pertussis* infection of HAE cells results in a reduction of the barrier function and disruption of mucociliary transport, closely representing hallmarks of natural pertussis disease^1^.

### Transcriptome analysis of *B. pertussis*-infected HAE cells

**Figure 3:**
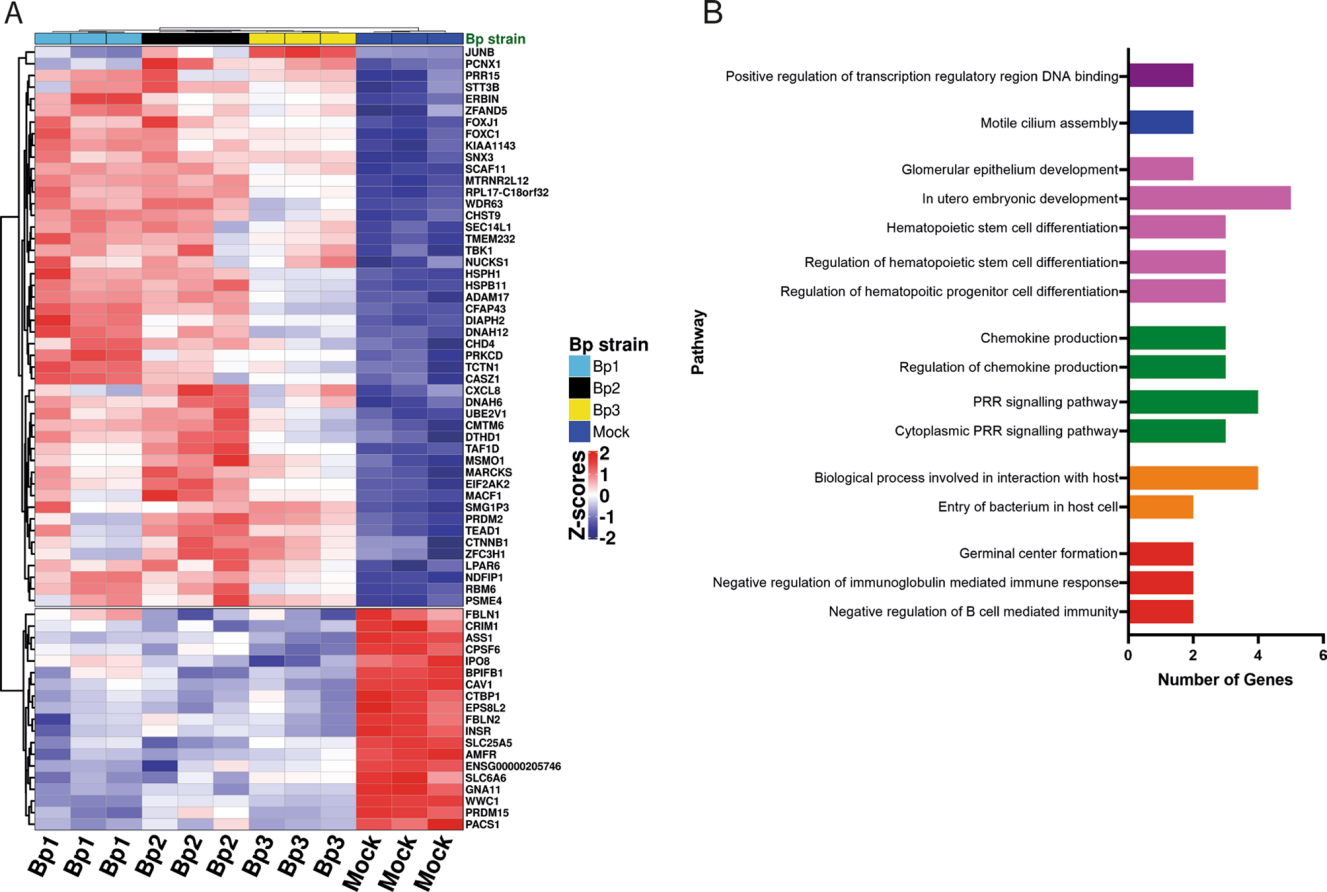
Transcriptome analysis of *B. pertussis*-infected HAE cells. (**A**) Heatmap representing hierarchical clustering of all up- or downregulated genes (padj < 0.05, rows; z-scores, scaled and centered per gene) after a 6 h stimulation with any of the three used *B. pertussis* strains per individual HAE donor (columns; Mock (dark blue), Bp1 (light blue), Bp2 (black) and Bp3 (yellow)). (**B**) GO enrichment analysis of *B. pertussis*-infected HAE cells representing number of upregulated genes present (x-axis) in enriched pathways (y-axis). Padj = 0.119 for all enriched pathways. Data are shown from transcriptome analysis from 3 individual donors.

To get a better insight into how HAE cells respond to *B. pertussis* infection we performed transcriptome analysis on HAE cells from three different donors infected for 6 h with three different *B. pertussis* strains at MOI 100. Analysis of differentially expressed genes (DEG) compared to Mock cells was focused on common DEG for all *B. pertussis*-infected HAE cells. This analysis revealed 48 commonly upregulated and 19 commonly downregulated genes (Fig. 3A, Fig S2), establishing a core DEG profile of these cells induced by *B. pertussis*. The 48 upregulated genes included several genes involved in cilia motility (*FOXJ1*, *CFAP43*, *DNAH12*, *DNAH6* and *HSPB11*) indicating that the cells respond to the *B. pertussis*-induced disruption of mucociliary clearance by increasing expression of these genes. Also, genes involved in innate immune activation such as *CXCL8*, the AP-1 transcription factor subunit *JUNB*, and *TBK1* involved in NF-κB activation were significantly upregulated after *B. pertussis* infection. No mucins or host defense peptides were significantly up- or downregulated after a 6 h *B. pertussis* infection.

To identify enriched pathways we performed GO enrichment analysis of the 48 core upregulated DEGs. This showed for *B. pertussis*-infected HAE an enrichment of various pathways associated with (hematopoietic) cell differentiation and tissue development (Pink), indicating active differentiation and development of the HAE cells (Fig. 3B). The pathway “Motile cilium assembly” is enriched (Blue), which is in accordance with the previously observed gene expression associated with cilia motility. The bacterium appears to actively interact with the epithelial cells (Orange, “Entry of bacterium in host cell”, “Biological process involved in interaction with host”). GO-enrichment analysis also revealed innate immune activation of the HAE, indicated by the enrichment of pathways associated with innate immunity (Green, “(Cytoplasmic) pattern recognition receptor (PRR) signaling”, “(Regulation of) chemokine production”).

### HAE immune activation

**Figure 4:**
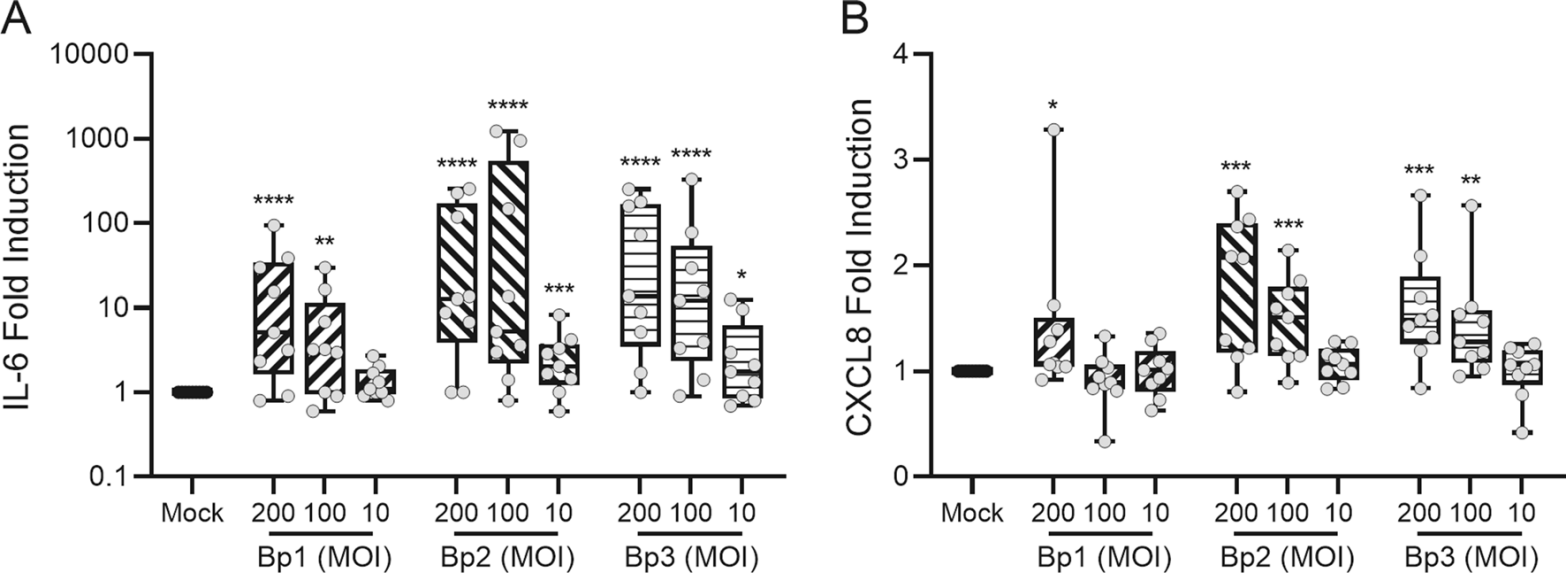
IL-6 and CXCL8 secretion by HAE cells stimulated with three different *B. pertussis* strains. (**A**) IL-6 and (**B**) CXCL8 secretion in the basal medium of HAE cultures after 22 h of stimulation. Data are represented as fold change of cytokine levels relative to that of the Mock-treated HAE cells. Box filling (stripes diagonal left, diagonal right and horizontal) represents the different *B. pertussis* strains used and individual dots are averages of triplicate measurements. The data shown are from 3 donors measured twice across 3 independent experiments. Significance represented relative to Mock-treated HAE cells. *p < 0.05, **p < 0.01, ***p < 0.001, ****p < 0.0001.

**Figure 5:**
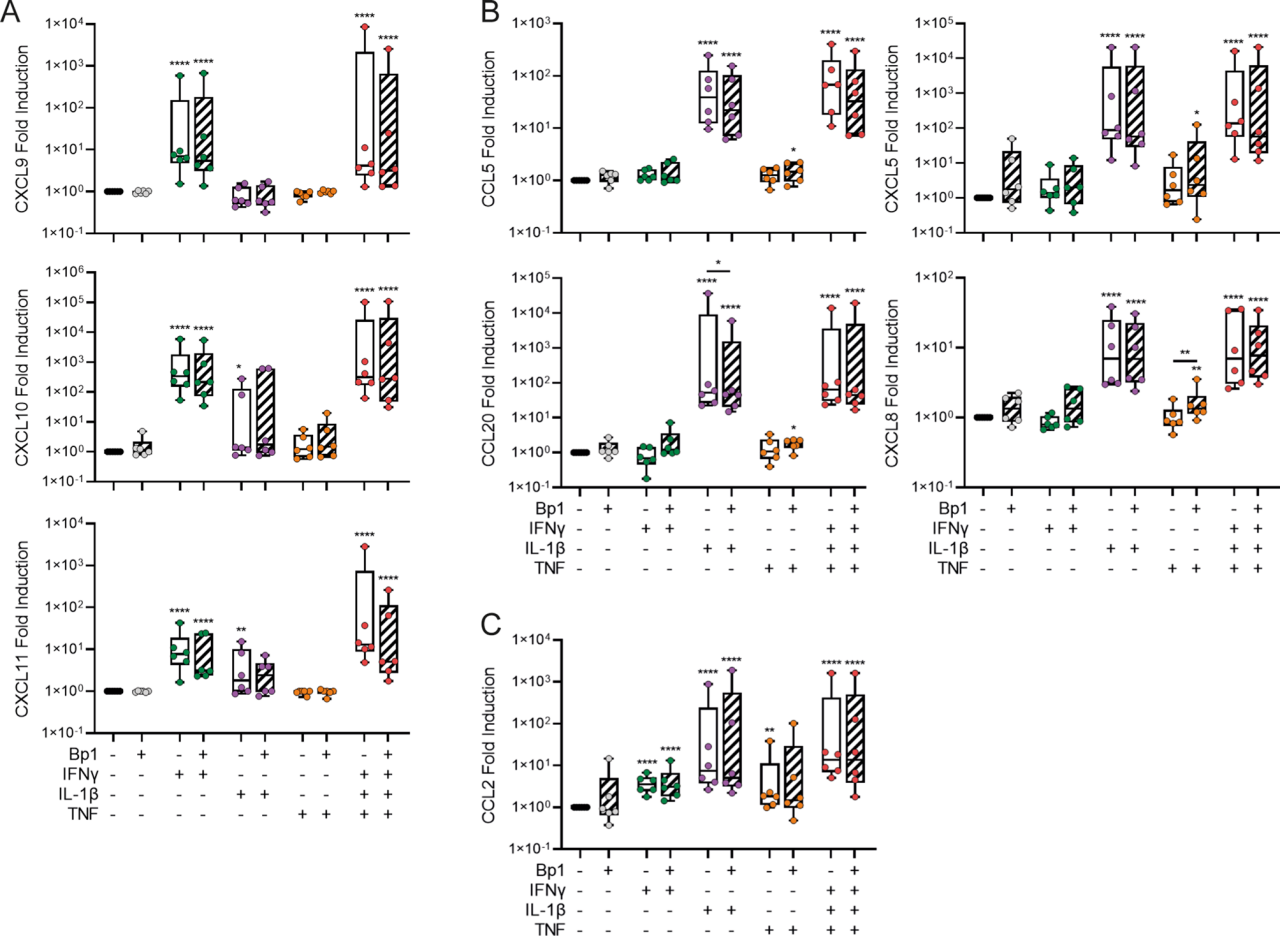
Cytokine-dependent chemokine secretion by HAE cells. (**A**) IFNγ-dependent chemokine secretion (CXCL9, CXCL10, CXCL11), (**B**) IL-1β-dependent chemokine secretion (CCL5, CCL20, CXCL5, CXCL8) and (**C**) IFNγ or IL-1β-dependent chemokine secretion (CCL2) by HAE cells after 22 h of stimulation in the absence of cytokines (black, grey) or in the presence of 10 ng/ml IFNγ (green), IL-1β (purple), TNF (orange) or all three cytokines (red) in the absence (open boxplot) or presence (diagonally striped boxplot) of Bp1 MOI 100. Data are represented as fold change of cytokine levels relative to that of the Mock-treated HAE cells. Individual dots are averages of triplicate measurements. The data shown are from 3 donors measured twice across 3 independent experiments. Significance represented relative to Mock-treated HAE cells, unless indicated otherwise. *p < 0.05, **p < 0.01, ****p < 0.0001.

To substantiate the RNA-seq data that suggest the induction of an innate immune response against *B. pertussis*, we analyzed cytokine and chemokine secretion into the basal medium of *B. pertussis*-infected HAE cells. IL-6 and CXCL8 increased dose-dependently after a 22 h incubation with any of the *B. pertussis* strains (Fig. 4). Since the human airway epithelium is able to secrete many more factors than the IL-6 and CXCL8 in response to respiratory pathogens as detected in our cultures^15,16^, we hypothesized that additional signals from immune cells may be required to induce the secretion of cytokines and chemokine by HAE cells in response to *B. pertussis*. To test this hypothesis we first added recombinant IFNγ, IL-1β and TNF to the HAE cells stimulated with Bp1 MOI 100. These cytokines are known to be secreted by immune cells in response to *B. pertussis*^5,10,17^. Addition of IFNγ induced CXCL9, CXCL10 and CXCL11 secretion into the basal medium of HAE cultures (Fig. 5A). Additionally, IL-1β induced low levels of CXCL10 and CXCL11 secretion (Fig. 5A), and high levels of CCL5, CCL20, CXCL5 and CXCL8 secretion (Fig. 5B). All cytokines were able to induce the secretion of CCL2 (Fig. 5C). Combination of all cytokines (IFNγ, IL-1β and TNF) induced secretion of all measured chemokines by the HAE. The additional presence of *B. pertussis* in these cultures did not significantly alter the secretion of any of the chemokines induced by IFNγ, IL-1β and TNF. Nevertheless, an increased trend in the levels of CCL5, CCL20, CXCL5 and CXCL8 was observed when TNF and *B. pertussis* were combined (Fig. 5B). None of the cytokines (IL-1β, TNF and IFNγ) were able to prevent the reduction in TEER upon infection with *B. pertussis* (data not shown).

### HAE immune cell crosstalk

**Figure 6:**
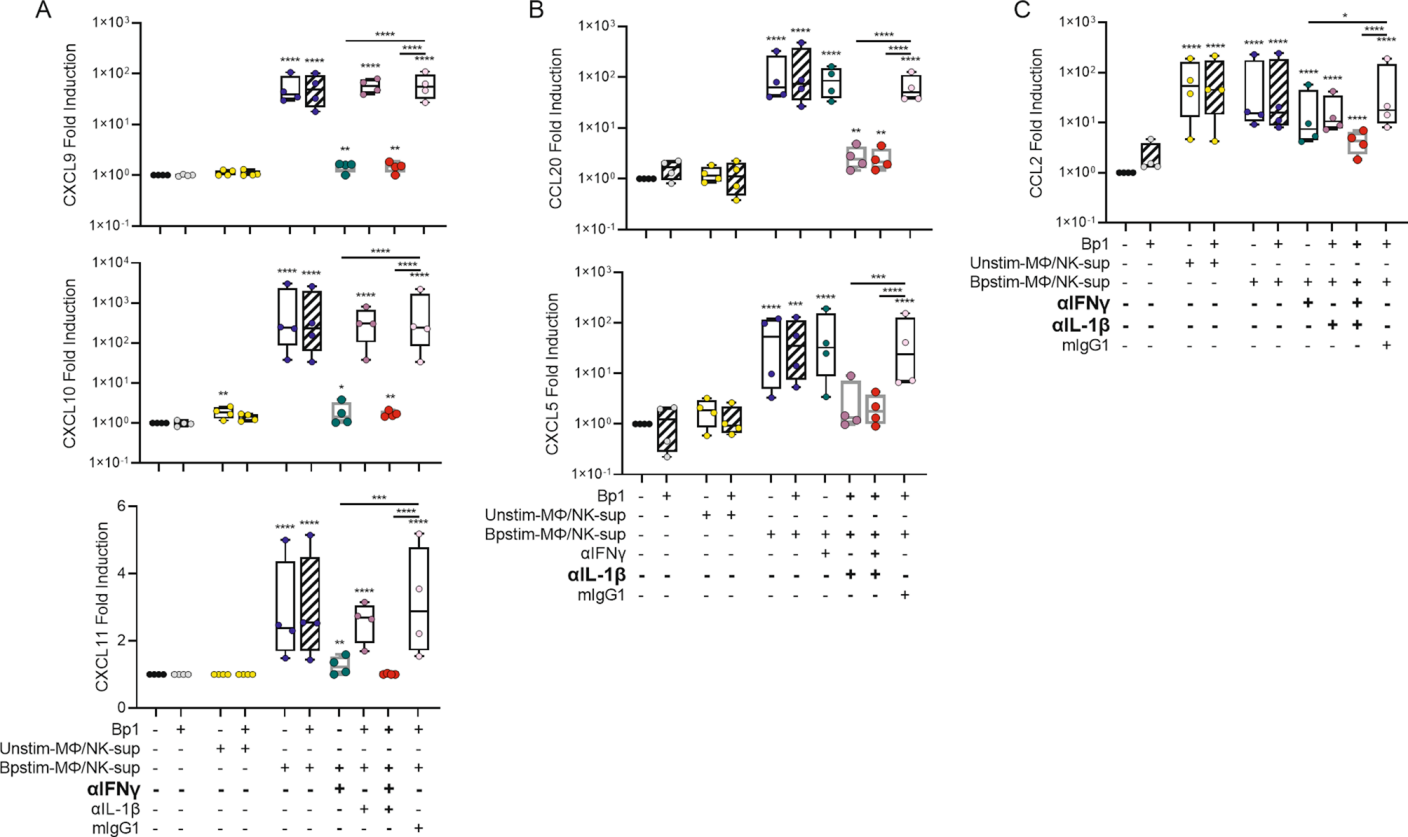
Chemokine secretion by HAE cells in response to stimulation with supernatants derived from *B. pertussis*- or Mock-treated macrophage/NK co-cultures. (**A**) IFNγ-dependent chemokine secretion (CXCL9, CXCL10, CXCL11), (**B**) IL-1β-dependent chemokine secretion (CCL20, CXCL5) and (**C**) IFNγ, IL-1β or TNF-dependent chemokine secretion (CCL2) by HAE cells after 22 h of stimulation in the absence of MΦ/NK-sup (black, grey) or in the presence of Unstim-MΦ/NK-sup (yellow) or Bpstim-MΦ/NK-sup in the absence of blocking antibodies (blue) or in the presence of αIFNγ (green), αIL-1β (purple), both (red), or the isotype control mIgG1 (pink). All these conditions were performed in the absence (open boxplot) or presence (diagonally striped boxplot) of Bp1 MOI 100. Data are represented as fold change of chemokine levels relative to that of the Mock-treated HAE cells. Individual dots are averages of triplicate measurements. The data shown are from 3 donors measured twice across 2 independent experiments. Unstim-MΦ/NK-sup = supernatant derived from unstimulated macrophage/NK cell co-cultures, Bpstim-MΦ/NK-sup = supernatant derived from *B. pertussis*-infected macrophage/NK cell co-cultures. Significance represented relative to Mock-treated HAE cells, unless indicated otherwise. *p < 0.05, **p < 0.01, ***p < 0.001, ****p < 0.0001.

To ascertain that also the more complex *B. pertussis* activation of immune cells can shape the innate HAE immune response to *B. pertussis*, we tested whether cytokines produced by *B. pertussis*-infected macrophages and NK cells were able to induce chemokine secretion by HAE cells. For this, we made use of supernatants derived from *B. pertussis*-infected macrophage/NK co-cultures (Bpstim-MΦ/NK-sup) or unstimulated macrophage/NK co-cultures (Unstim-MΦ/NK-sup) as previously described by our group^17^. Bpstim-MΦ/NK-sup contained a wide variety of cytokines and chemokines, which were absent or present in lower concentrations in the Unstim-MΦ/NK-sup. CCL2 and CXCL8 were present in similar quantities in the Unstim- and Bpstim-MΦ/NK-sup (Fig S2). HAE cell exposure to Bpstim-MΦ/NK-sup resulted in increased secretion of CXCL9, CXCL10, CXCL11, CCL2, CCL5, CCL20 and CXCL5 compared to these cells exposed to Unstim-MΦ/NK-sup (Fig. 6). Simultaneous stimulation with *B. pertussis* did not enhance nor diminish the chemokine secretion. CCL2 was also detected in the basal medium of Unstim-MΦ/NK-sup stimulated HAE cultures, however, this is likely due to the high concentrations of CCL2 present in the Unstim-MΦ/NK-sup. To learn more about which factors secreted by immune cells mainly contributed to the HAE cell response, we blocked IFNγ in Bpstim-MΦ/NK-sup by neutralizing antibodies. This almost completely abrogated CXCL9, CXCL10 and CXCL11 secretion by the HAE cells (Fig. 6A). On the other hand, IL-1β-neutralizing antibodies significantly inhibited CCL20 and CXCL5 secretion (Fig. 6B) and only when blocking both IL-1β and IFNγ activity, lower levels of CCL2 secretion were observed (Fig. 6C). These data strongly suggest that IFNγ and IL-1β are required for HAE cells to initiate the robust secretion of chemokines. This highlights the importance of macrophage and NK cell activation by *B. pertussis* for the induction of the HAE immune response to this pathogen.

### HAE chemokine secretion induces monocyte migration

**Figure 7:**
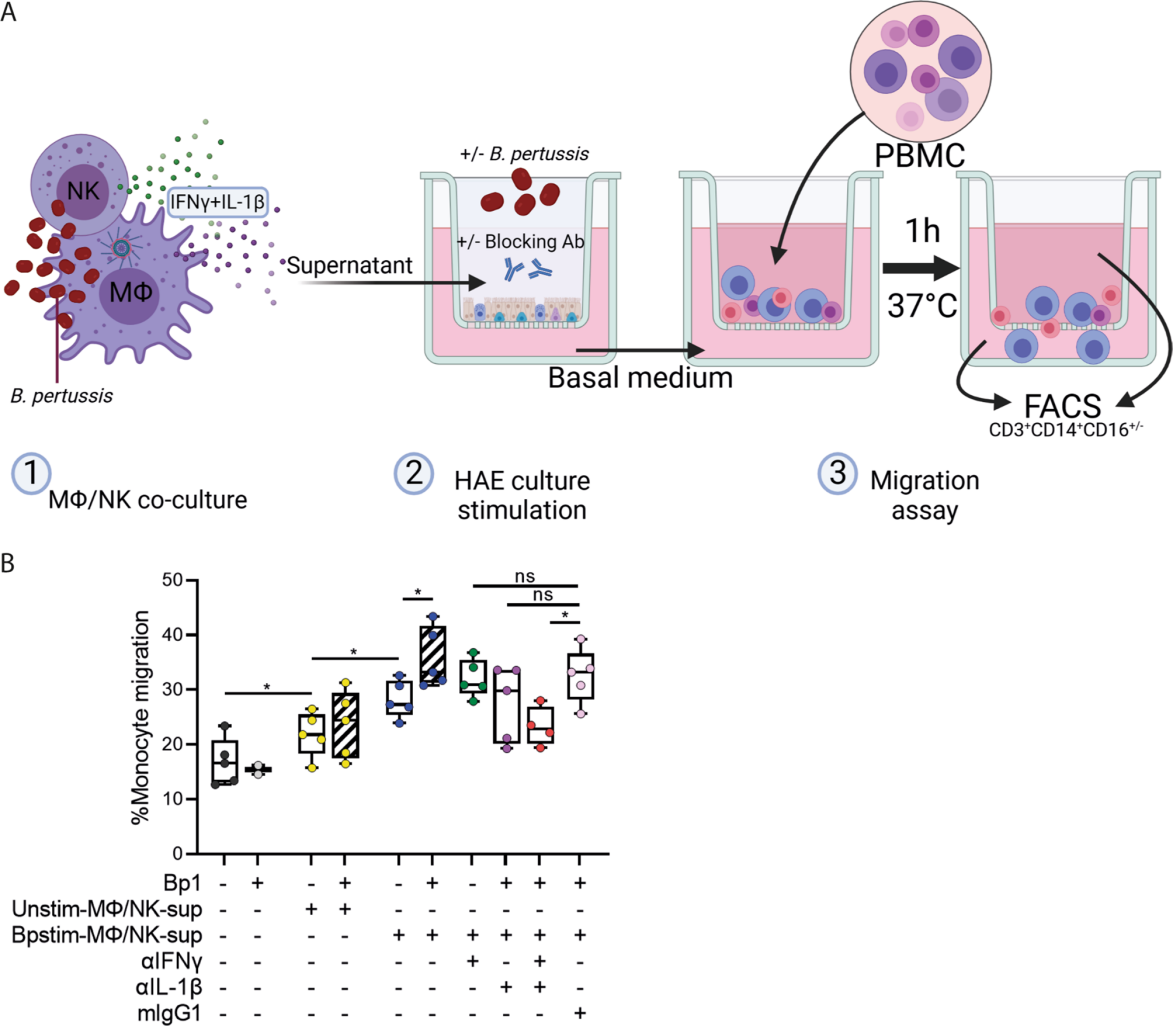
Monocyte migration in response to stimulated HAE culture basal medium. (**A**) Graphical representation of the PBMC migration experiment. Image created with BioRender.com (**B**) % of monocyte migration in response to the basal medium derived from HAE cultures after 22 h of stimulation in the absence of MΦ/NK-sup (black, grey) or in the presence of Unstim-MΦ/NK-sup (yellow) or Bpstim-MΦ/NK-sup in the absence of blocking antibodies (blue) or in the presence of αIFNγ (green), αIL-1β (purple), both (red), or the isotype control mIgG1 (pink). All these conditions were performed in the absence (open boxplot) or presence (diagonally striped boxplot) of Bp1 MOI 100. Data are represented as the amount of CD3^-^CD14^+^CD16^+/-^ cells in the basal compartment relative to the total amount of CD3^-^CD14^+^CD16^+/-^ cell in the basal and apical compartment. Individual dots are averages of triplicate measurements. Representative data shown of HAE basal medium derived from 4 donors from 4 independent experiments used in 2 independent PBMC migration assays. *p < 0.05.

To determine whether the factors secreted by HAE cells are able and sufficient to induce immune cell migration, we performed a PBMC migration assay. For this, we made use of HAE basal medium after stimulation with either Unstim-MΦ/NK-sup or Bpstim-MΦ/NK-sup as described in Fig. 6. This basal medium was transferred to the basal compartment of an empty transwell system and PBMCs were added to the apical chamber. After a one-hour incubation the cells present in the apical and basal chamber were analyzed by flow cytometry (Fig. 7A). This revealed significant monocyte migration when basal medium of Bpstim-MΦ/NK-sup stimulated HAE cultures was used as chemoattractant (Fig. 7B). The monocyte migration was further enhanced when basal medium from the HAE cultures that had simultaneously been stimulated with *B. pertussis* and Bpstim-MΦ/NK-sup was used. When basal medium was used where both IL-1β and IFNγ activity were blocked in the Bpstim-MΦ/NK-sup prior to adding it to the HAE cells, monocyte migration was significantly reduced compared to the isotype control. These results indicate that immune factors produced by HAE cells in response to both IL-1β and IFNγ are capable to induce monocyte migration.

## Discussion

*Bordetella pertussis* is an exclusive human respiratory pathogen that infects the ciliated airway epithelium. Many virulence factors from this pathogen that contribute to infection and disease have been investigated in great detail (reviewed in^18^). Much less is known about how the human airway epithelium (HAE) functions as an immunological barrier during *B. pertussis* infection. In the present study, we used primary in vitro differentiated bronchial/tracheal human epithelial cells as an infection model. The cell culture consistently yielded HAE that was covered with cilia, produced mucus and formed a tight barrier. HAE cells kept at the air–liquid interface are an ideal in vitro model to investigate the response of these cells to *B. pertussis* as it closely represents the natural infection niche.

Findings from this study indicate that infection of HAE cells with various naturally circulating *B. pertussis* strains disrupted the integrity of the HAE barrier. This pathological effect resembles the toxic outcome of *B. pertussis-*derived tracheal cytotoxin (TCT) on the airway epithelium of both hamsters^6,19,20^ and humans^7,21^. Our finding that all tested *B. pertussis* strains completely abrogated the ability of the HAE cells to transport beads across their apical surface is consistent with the loss in mucociliary clearance by ciliated epithelium which is a hallmark of natural *B. pertussis* infection. Reduced mucociliary clearance has previously been attributed to a reduction of the amount of ciliated cells rather than a reduction in ciliary beat frequency^7^. Although we have not counted the amount of ciliated cells, we did observe that after *B. pertussis* infection the HAE cultures still contained beating ciliated cells but were nonetheless unable to transport beads (Movie S4).

As a first step to elucidate the HAE response to *B. pertussis* infection, we performed RNA-seq and compared the transcriptomes of *B. pertussis*-infected and non-infected HAE cells. We observed a large variation in the amount of DEGs induced by the different *B. pertussis* strains (Fig S1). However, this was most likely due to the low amount of donors available for the RNA-seq and does not necessarily reflect actual differences between the strains. Whether these differences are due to the variation among the stains which may differently express other antigens besides pertactin^22,23^ could be investigated in future studies using more *B. pertussis* strains per group. To make sure we analyzed genes differently expressed after *B. pertussis* infection we only included the genes commonly up- or downregulated after incubation with any of the tested *B. pertussis* strains. Analysis of *B. pertussis*-infected HAE cells revealed immunological activation as indicated by the upregulation of genes involved in PRR signaling and chemokine production. However, analysis of secreted cytokines and chemokines in the basal medium of HAE cultures after *B. pertussis* infection revealed the secretion of only IL-6 and CXCL8 and no other chemokines. IL-6 is a proinflammatory cytokine which has a wide variety of effects on many different branches of the immune system^24^. CXCL8 is a chemokine responsible for attracting neutrophils to the site of infection and activating them^25^. IL-6 and CXCL8 transcription is regulated by NF-κB and AP-1^26–28^. Both these transcription factors were significantly upregulated in *B. pertussis*-infected HAE. However, no other cytokines or chemokines regulated by NF-κB or AP-1^29^ were found to be secreted by *B. pertussis*-infected HAE. While both IL-6 and CXCL8 were secreted by *B. pertussis*-infected HAE cells, only *IL8*, encoding for CXCL8, was significantly upregulated after a 6 h incubation. Another study, which analyzed the transcriptional profile of *B. pertussis-*infected undifferentiated BEAS-2B cells, a transformed human bronchial epithelial cell line, after a 3 h incubation did find significant upregulation of both *IL6* and *IL8*^30^. This could indicate that *IL6* upregulation already occurred in HAE cells by the time we performed RNA-seq, which was at 6 h after incubation with *B. pertussis*. Additionally, while they found *IL1B* to be upregulated in BEAS-2B cells, we did not observe any increased expression or secretion of IL-1β (data not shown) by HAE cells after *B. pertussis* infection. As IL-1β secretion is regulated post-transcriptionally^31^, the lack of IL-1β secretion does not exclude the upregulation of *IL1B* before our transcriptome analysis was performed.

We were surprised to find only IL-6 and CXCL8 secretion by *B. pertussis*-infected HAE because it is known that *B. pertussis* infection leads to massive immune cell infiltration into the lungs^1,10^, which requires the production of chemokines. Additionally, our findings indicate that *B. pertussis* did not have any enhancing or inhibitory effect on the cytokine-induced chemokine secretion by HAE cells. The lack of strong immunological activation of the HAE by *B. pertussis* may be due to strong inhibitory properties of *B. pertussis* virulence factors. Using murine models for pertussis, the virulence factor pertussis toxin has been shown to inhibit neutrophil recruitment to the lungs and to actively suppress chemokine expression^32^^,^^33^. However, in those studies the authors analyzed the effect of pertussis toxin on chemokine expression in whole lungs of *B. pertussis*-infected mice and on the MH-S murine alveolar macrophage cell line but have not looked at the effect on the airway epithelium itself. Future studies should elucidate whether the inhibitory effect of pertussis toxin on chemokine production and cell recruitment is due to inhibition of other immune cells present in the lungs or by also targeting the airway epithelial cells. Another study showed the ability of recombinant adenylate cyclase toxin (ACT), a virulence factor produced by *B. pertussis*, to inhibit IL-17A induced CXCL8 secretion by HAE cells^34^. We showed that the live naturally circulating *B. pertussis* strain used in this study, which has previously been shown to produce ACT^22^, was unable to inhibit IL-1β-induced CXCL8 secretion. Future studies should investigate whether live *B. pertussis* is able to inhibit IL-17A-induced chemokine secretion by the HAE and whether this is dependent on *B. pertussis*-derived ACT.

In the natural situation, HAE closely interacts with immune cells^8^, therefore, we hypothesized that cytokines secreted by immune cells present at the epithelial lining were essential to fully activate the airway epithelial cells. Indeed, our results indicate that intercellular communication between HAE and local innate immune cells namely, macrophages and NK cells, is essential for the secretion of a wide variety of chemokines by the HAE. Macrophages and dendritic cells are the first innate immune cells that sense and respond to *B. pertussis*^10^ by secreting cytokines such as, IL-1β, TNF and IL-6^5,10^, and together with NK cells also produce IFNγ^17^. Exposure of HAE to recombinant IL-1β and IFNγ resulted in robust chemokine secretion. However, exposure to the more complex supernatant of *B. pertussis*-infected macrophages and NK cells also resulted in the secretion of a wide variety of chemokines by the HAE. Especially IL-1β and IFNγ derived, among others, from *B. pertussis*-infected macrophages and NK cells, induced the production of chemokines by HAE cells. As both early IL-1β and IFNγ secretion in response to *B. pertussis* were shown to depend on NLRP3 inflammasome activation in macrophages^17,35^ this also highlights the important role of inflammasome activation in shaping an inflammatory response to *B. pertussis* in the HAE.

In the present study, we showed that immune factors produced by cytokine-stimulated HAE cells were able to induce monocyte migration and that this was dependent on both IL-1β and IFNγ stimulation of the HAE cells. Since CCL2 in known to attract monocytes^36^ and both IL-1β and IFNγ induced the secretion of CCL2, this chemokine is likely responsible for the observed monocyte migration. However, since monocytes express a wide variety of chemokine receptors which are targeted by a broad range of chemokines^37^, future studies should investigate the contribution of different chemokines to monocyte migration. Monocyte migration induced by basal medium derived from Bpstim-MΦ/NK-sup stimulated HAE cultures was increased if the HAE cells were simultaneously stimulated with *B. pertussis*. Since we did not find any increased chemokine production by cytokine-stimulated HAE cells in the presence of *B. pertussis* it appears that unmeasured chemokines or other undetected factors, such as host defense peptides^38^, are responsible for the increased monocyte migration. Here, we focused on monocyte migration because these innate cells are among the first to be recruited to the lungs during *B. pertussis* infection^10^ and after their differentiation to macrophages they secrete IL-1β and IFNγ both required to enhance the immune response to this pathogen.

**Figure 8:**
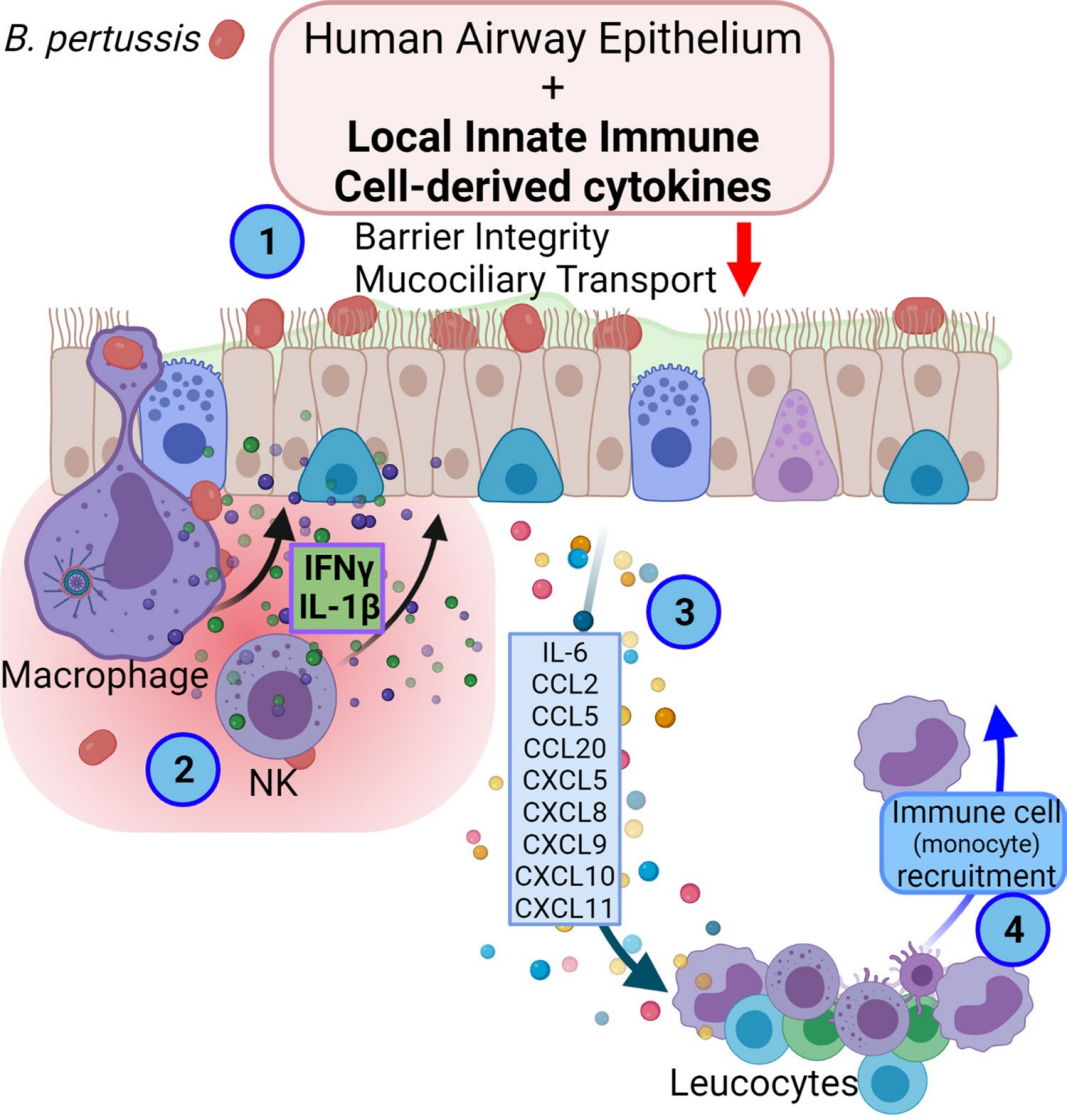
Graphical representation of the human airway epithelium response to *B. pertussis*. Upon infection of the HAE, *B. pertussis* disrupts the epithelial barrier integrity and mucociliary clearance (1). Local innate immune cells such as macrophages and NK cells sense the presence of *B. pertussis* and produce cytokines including, IFNγ and IL-1β (2). These cytokines stimulate the HAE to produce a wide variety of chemokines (3) which are required for the recruitment of immune cells to the site of infection (4) amplifying local inflammation. Image created with BioRender.com.

Altogether, we show that primary HAE cultures are an excellent model to investigate the early events of *B. pertussis* infection. Using this in vitro model we could mimic disruption of epithelial barrier integrity, reduction of mucociliary clearance, immune activation and immune cell infiltration, which are all hallmarks of pertussis disease^1^. We identified IL-1β and IFNγ as essential immunological factors for a robust HAE response against *B. pertussis*. Our data suggests that the airway epithelium on its own has a limited immunological response to *B. pertussis* and that for a broad response, local innate immune cells are necessary (Fig. 8). In other words, the cytokines secreted by these local immune cells upon infection with this pathogen stimulate the airway epithelium to produce different chemokines which are required for the recruitment of additional immune cells enhancing local inflammation. This highlights the importance of local intercellular communication in the defense against *B. pertussis* infection and may add to the design of next generation pertussis vaccines that aim to prevent not only disease but also airway colonization by the bacterium.

## Materials and methods

### Bacterial strains and growth conditions

In this study we used three *B. pertussis* strains isolated from Dutch pertussis patients: two isolated in 2016 (Bp1 and Bp2) and another isolated in 1998 (Bp3), Bp2 was deficient for the virulence factor pertactin. All strains were selected based on the grouping described by Kroes et al.^22^. In short, all strains have comparable genotypes (pertussis toxin (ptx) promoter 3, pertactin type 2, ptxA type 1, fimbriae 2/3 type 1) and only differ in pertactin expression or year of isolation. To ensure the consistent use of the *B. pertussis* strains at a logarithmic growth phase, flash frozen cultures were prepared as previously described^17^ and used for all experiments.

### Human airway epithelium air–liquid culture

Primary human bronchial/tracheal epithelial cells from four healthy donors (Lonza, catalog#: CC-2540) were expanded in serum-free PneumaCult-Ex medium supplemented with hydrocortisone (480 ng/ml), penicillin (100 U/ml) and streptomycin (100 μg/ml) at 37 °C and 5% CO_2_. ReagentPack Subculture Reagents (Lonza) were used to passage the cells. Before reaching passage 4, a total of 50,000 cells were seeded into 6.5 mm transwell plates with 0.4 μm pore polyester membrane inserts (Corning Inc) that were pre-coated with Collagen I Rat Tail Protein (30 μg/ml) for 45 min at 37 °C. After 2–4 days of submerged culture in complete PneumaCult-Ex medium (500 μl in basal and 100 μl in apical compartment) the apical medium was removed and the basal medium was replaced with 500 μl serum-free PneumaCult-ALI medium supplemented with hydrocortisone (480 ng/ml), Heparin (4 μg/ml), penicillin (100 U/ml) and streptomycin (100 μg/ml), creating an air–liquid interface. These HAE air–liquid cultures were differentiated for 6–8 weeks towards a pseudostratified epithelial cell layer. PneumaCult-ALI medium in the basal compartment was refreshed every 2–3 days and after 2 weeks the apical surface was washed every week with 200 μl PBS for 5 min at 37 °C.

### HAE infection assays

Two days prior to use in experiment, medium of the HAE cells was replaced by PneumaCult-ALI medium without antibiotics. HAE cells were inoculated with live *B. pertussis* at the indicated multiplicity of infection (MOI) which was calculated relative to the amount of cells seeded onto the insert. Additionally, HAE cells were treated with either recombinant human IFNγ, IL-1β, TNF-α (10 ng/ml), and anti-human IFNγ, anti-human IL-1β or the isotype control mouse IgG1 (10 μg/ml). All suspensions were prepared in PneumaCult-ALI medium without antibiotics and HAE cells were exposed in a total of 50 μl in the apical compartment for 22 h at 37 °C and 5% CO_2_. Polymyxin B was added to a concentration of 100 μg/ml to all collected supernatants and incubated for 30 min at 37 °C to kill any live bacteria present before storage at − 80 °C. Conditioned macrophage/NK co-culture supernatants were prepared as previously described^17^. Briefly, monocytes were isolated from blood of healthy donors and differentiated to macrophages using GM-CSF. Autologous NK cells were added to the monocyte-derived macrophages in a 1:1 ratio and these co-cultures were stimulated with *B. pertussis*. After a 20 h stimulation supernatants were collected and filtered using a 0.22 μm filter to remove any bacteria.

### Transepithelial electronical resistance (TEER) measurement

To measure TEER of the HAE cultures, 200 μl of PBS was added to the apical chamber and TEER was immediately measured using a Millicell ERS-2 Volt-ohm meter. To prevent contamination of the supernatants when measuring TEER after infection assays, the transwell inserts were first transferred to a new 24-well plate containing fresh PneumaCult-ALI medium (500 μl) in the basal compartment after which PBS (200 μl) was added to the apical compartment prior to TEER measurement. Background TEER, determined by measuring TEER of an empty insert, was subtracted from the TEER values.

### Fluorescence microscopy

HAE cells were differentiated for 7 weeks and then fixated in paraformaldehyde (4%) for 15 min at 37 °C. Cells were permeabilized with Triton X-100 (0.1%) for 5 min, blocked with BSA (1%), normal serum block (2%, Goat-serum, Biolegend) and Triton X-100 (0.05%) in PBS for 30 min. Subsequently, the cells were stained with mouse anti-β-tubulin-IV (30 μg/ml, Clone ONS.1A6, Sigma, staining ciliated cells) and rabbit anti-Muc5AC (3.37 μg/ml, Clone EPR16904, Abcam, staining mucus producing cells), or mouse anti-ZO-1 (10 μg/ml, Clone ZO1-1A12, staining tight junctions) for 1 h at 37 °C. For the secondary staining donkey anti-rabbit Alexa Fluor 555 (10 μg/ml, for Muc5AC staining, Biolegend), goat anti-mouse DyLight 488 (10 μg/ml, for β-tubulin-IV straining, Biolegend) or DyLight 649 (for ZO-1 staining, Biolegend) was added for 1 h at 37 °C. Nuclei were stained with DAPI (300 nM) for 10 min at 37 °C. The polyester membrane containing the cells was excised from the transwell insert, placed on a microscopy slide, and embedded in ProLong™ Diamond Antifade Mountant (Invitrogen). Images were acquired using the Leica Dmi8 microscope with a 100 × objective and Leica DFC7000 GT camera using LAS X 3.4.2 software.

### Hematoxylin and eosin (HE) staining

HAE cells that had been differentiated for 7 weeks were fixated in PFA (4%) for 4 h, dehydrated, embedded in paraffin, and sectioned. Six μm sections were deparaffinized and stained according to standard HE procedures. Images were made using the Leica Dmi8 microscope with a 40X objective and Leica MC190HD camera using LAS X 3.4.2 software. Images were cropped and a scalebar was added with Adobe Photoshop version 19.1.19.

### Cilia movement and bead tracking

Inserts were washed with PBS (150 μl) for 5 min at 37 °C. Next, 50,000 fluorescent Sky Blue beads (Spherotech) of 1.7–2.2 μm diameter suspended in PBS (25 μl) were added to the apical compartment and beads were allowed to settle on the epithelial cells for 10 min at 37 °C. Excess PBS was removed from the apical compartment and bead movement was recorded at 37 °C in the Y5 channel of a Leica DMi8 microscope with a Leica DFC7000 GT camera by making a 30 s movie containing 149 frames. Two or three movies were recorded per well. Because bead speed in the center of the insert can differ from the speed near the edges of the insert all movies were recorded at several locations exactly one field of view from the edge. Leica LAS X 3.4.2 software with LAS X time-lapse extension was used for image analysis. Beads were automatically detected with a minimal size of 10 pixels. Particle tracks were generated by the software using a link range of 2 and a max displacement of 20–40 depending on the speed of the beads. Tracks of less than 8 concessive frames were removed from the analysis. The location of each bead at a certain time point was defined by a pixel centroid X axis (cX) and a pixel centroid Y axis (cY) position. Per group, all the locations were centered around 0 (to be able to overlap them) and were plotted with the aid of *ggplot2* R package^39^. For visualization purposes, only the top 10 lengthiest and most representative bead tracks (set of locations of a certain bead through time, indicated by arrows) from the Mock (unstimulated HAE) and *B. pertussis* infected HAE cells were represented. A local polynomial regression under a Locally Estimated Scatterplot Smoothing method of all the tracks (function *stat_smooth()* from *ggplot2* R package) was generated. This provided a general trend of all tracks in a single modeled track (black line overlapping all the trajectories) with the corresponding confidence interval (grey zone surrounding the black line).

### Chemokine and cytokine analysis

The concentration of cytokines and chemokines in the basal medium of HAE cultures and in the conditioned macrophage/NK cell co-culture supernatants were determined using the Human Proinflammatory Chemokine Panel 1 and Human Inflammation Panel 1 LEGENDplex kits (Biolegend) according to the manufacturer’s instructions. LEGENDplex beads were measured on a FACSCanto II cell analyzer and data were analyzed using the online tool provided by Biolegend at legendplex.qognit.com. The concentration of CXCL8 in the basal medium of HAE cultures was measured using the IL-8 Ready-SET-Go ELISA kit (ThermoFisher) according to the manufacturer’s instructions.

### RNA isolation, sequencing, analysis and data processing

Total RNA was extracted from HAE cells exposed to medium only, and after a 6 h incubation with *B. pertussis*. Library preparation and RNA sequencing were performed as previously described^22^. FASTQ files were aligned to the Homo sapiens reference genome (GRCh38) and most recent transcript annotations using *kallisto* (v0.46.1)^40^. Raw counts were filtered for low count genes, eliminating those targets with less than 1 count in at least 5 samples. Filtered count distribution was normalized with the *EDAseq* R package^41^ by the use of *betweenLaneNormalization()* function and the full quantile method. The suitability of this global normalization method was assessed and confirmed by *quantro* R package^42^.

*Remove Unwanted Variation using Residuals* (*RUVr*) from *RUVseq* R package^43^ was employed to obtain the covariates that correct for Donor and other technical bias in the downstream analysis. Two parameters were needed to be provided for that purpose: residuals and parameter “*K*”. Deviance residuals were obtained from a fitted *Genewise Negative Binomial Generalized Linear Model* (*glmFit()* function from *edgeR* R package^44^). The model design was ~ *Cell type*, corresponding to the different experimental groups: unstimulated HAE (Mock) and HAE stimulated with any of the three strains used in the study. Parameter “*K*” was set at 4, since it is the number of significant surrogate variables detected by *svaseq()* function of *sva* R package^45^, when using the same model design as for *glmFit*.

### Differential gene expression analysis and pathway enrichment analysis

The DEG lists was obtained with *DeSeq2* R package^46^ under standard parameters, for the comparisons Mock vs Bp1, Mock vs Bp2 and Mock vs Bp3. The model design was composed by the 4 covariates obtained in the *RUVr* correction (function *pData()*) and the ~ *Cell type* parameter described above, using as input the filtered normalized counts. Genes were considered significantly expressed if they showed an adjusted p-value (*Bonferroni-Hochberg* multiple testing (padj)) lower than 0.05 and a Fold Change > 1.2.

Venn diagrams were produced with *VennDiagram* R package^47^, giving as input all the DEG lists from the different comparisons.

Heatmap to represent the selected DEGs were generated with the aid of *ComplexHeatmap* R package^48^. Dendrogram trees of the heatmap were obtained by hierarchical clustering (*Ward’s D2* method) of *spearman* correlation gene distances.

Gene Ontology (GO) enrichment of the selected DEGs was performed and plotted by using the functions *enrichGO()* and *barplot()* respectively of the *clusterProfiler* R package^49^. As input, only the common up-regulated and down-regulated genes in the comparison of the 3 strains vs Mock were used.

### Monocyte migration assay

Monocyte migration was determined by seeding 500,000 PBMC, isolated from blood of healthy donors using lymphoprep gradient centrifugation, in the apical chamber of a Corning HTS Transwell 96 well permeable support with 3.0 μm pore polycarbonate membrane. One hundred microliters of undiluted basal medium derived from HAE cells was added to the basal compartment of the transwell system and the plates were incubated for 1 h at 37 °C and 5% CO_2_. After incubation, cells from the apical and basal compartment were collected separately and both compartments were washed extensively with PBS. Cells attached to the bottom of the membrane were detached by incubating for 10 min at 37 °C with Accutase (100 μl) in the basal compartment. Detached cells were added to the cells collected from the basal compartment. Marker expression was determined with a LSRFortessa X-20 (BD) and data were analyzed using FlowJo software version 10.6.2. The percentage of monocyte migration was calculated by determining the abundance of CD3^−^CD14^+^CD16^+/−^ cells in the basal compartment relative to the total amount of CD3^−^CD14^+^CD16^+/−^ cells in the apical and basal compartment.

### Ethics statement

This study was conducted according to the principles described in the Declaration of Helsinki. Buffy coats were provided by the Sanquin blood supply. For the collection of blood samples and subsequent analyses, all blood donors provided written informed consent. Blood samples were processed anonymously and the research goal, primary cell isolation, required no review by an accredited Medical Research Ethics Committee, as determined by the Dutch Central Committee on Research involving human subjects.

### Statistical analysis

Data were subjected to a Shapiro–Wilk normality test and when passed subjected to a two-tailed paired t-test using the GraphPad Prism version 9.1.0 software and corrected for multiple testing using the Bonferroni-Holm method in RStudio.

For the ciliary movement, µm/s were used for representation and statistics. In the rest of the analysis of cytokine secretion, the raw cytokine concentrations were divided by the triplicate average in the medium condition of the corresponding donor, to normalize for the donor effect and to obtain a relative change to that condition (Fold Induction). Having these values as input, the permutation based non-parametric Exact Wilcoxon-Mann–Whitney test was applied for the different comparisons of interest. All the *p*-values were corrected for multiple testing by the Bonferroni-Hochberg method (padj). For representation purposes, the triplicate average per donor of the normalized data was used.

Effect sizes (ES) were calculated, providing Cliff’s delta^50^ (function *orddom()* from *orddom* R package^51^). The descriptors for Cliff’s delta are: small, < 0.28; medium, < 0.43; large, >  = 0.43^52^. All comparisons with both a padj < 0.05 and ES medium or higher are considered as biologically relevant and highlighted in the present work.

## Supplementary Information

Supplementary Video 1.

Supplementary Video 2.

Supplementary Video 3.

Supplementary Video 4.

Supplementary Information 1.

## Data Availability

All RNA-seq data is available in the GEO database, accession number: GSE182807 (reviewer token: ehwbyyukjxgdnyx).
