# Supplementary material for: Bordetella pertussis-infected innate immune cells drive the anti-pertussis response of human airway epithelium
Source: Sci Rep. 2022 Mar 7;12:3622. doi: 10.1038/s41598-022-07603-8 (PMC8901624; doi:10.1038/s41598-022-07603-8)
Supplement: Supplementary file 5 — Supplementary Information 1. [file 41598_2022_7603_MOESM5_ESM.pdf]

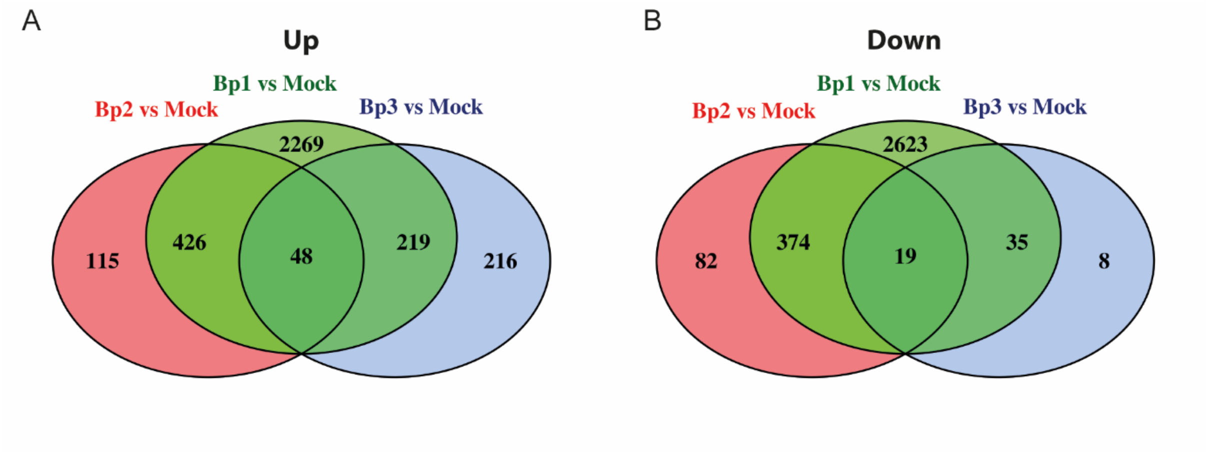

Supplemental Figure 1. **Venn Diagrams.** Venn diagrams of the DEGs (A) Up- or (B) Down-regulated in the Bp1, Bp2 or Bp3 vs Mock-treated HAE cells.

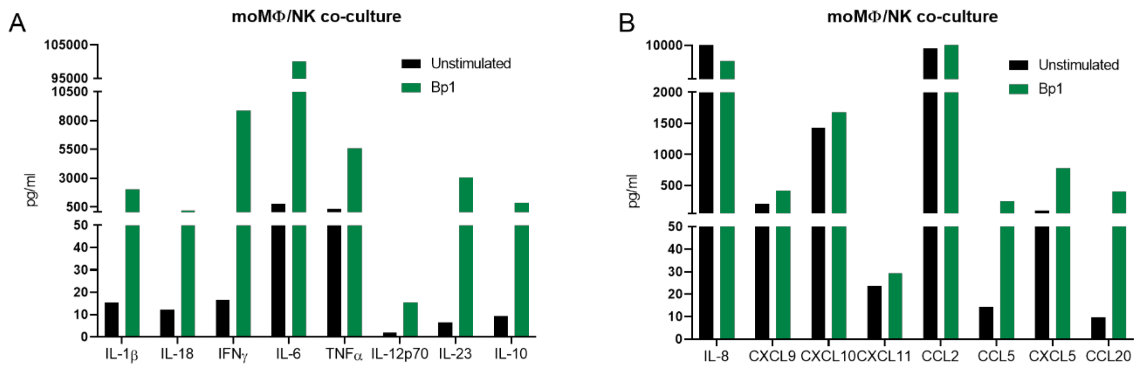

Supplemental Figure 2. **MΦ/NK co-culture cytokine/chemokine legendplex.** (A) Cytokine and (B) chemokine secretion of unstimulated or *B. pertussis*-infected MΦ/NK co-cultures.

Supplemental Movie 1. **Mock-treated HAE cells bead tracking.** Movie of fluorescent bead movement in Mock-treated HAE cells. Recorded in the Y5 fluorescence channel.

Supplemental Movie 2. **Mock-treated HAE cells cilia beating.** Movie of beating cilia in Mock-treated HAE cells recorded in the brightfield channel.

Supplemental Movie 3. ***B. pertussis*-infected HAE culture bead tracking.** Movie of fluorescent bead movement in HAE cultures after a 22 hour stimulated with Bp1 MOI 100. Recorded in the Y5 fluorescence channel.

Supplemental Movie 4. ***B. pertussis*-infected HAE culture cilia beating.** Movie of beating cilia in HAE cultures after a 22 hour stimulation with Bp1 MOI 100. Recorded in the brightfield channel.
